# Landscaping Agricultural and Animal Husbandry Production Park Using Lightweight Deep Reinforcement Learning under Circular Symbiosis Concept

**DOI:** 10.1155/2022/8410996

**Published:** 2022-06-02

**Authors:** Yiwen Cui

**Affiliations:** Department of Art and Design, Shaanxi Fashion Engineering University, Xi'an City 710000, China

## Abstract

The paper intends to optimize the landscape of the agricultural and animal husbandry (AG and AH) production park using the deep reinforcement learning (DRL) model under circular symbiosis. Therefore, after reviewing the relevant literature, decision tree evolutionary algorithm, and ensemble learning criteria, this paper studies and constructs the circular symbiotic industrial chain. Then, an experiment of landscaping the park and optimizing the production is made with full consideration of practical institutions. Finally, the numerical results show that the yield of several crops has been significantly improved after the landscape optimization by the proposed DRL model. Remarkably, the increase in rice yield is the most prominent. The yield of rice and wheat was about 12 kg before optimization and 18 kg after DRL model optimization, which has increased by 6 kg. This research has important reference value for improving the output efficiency of AG and AH products.

## 1. Introduction

The booming domestic economy has elevated material well-being mostly at the cost of environmental damage and pollution, especially in urban constructions. For example, trillions of tons of garbage are disposed of by urban activities to the ecological environment every day, with some extremely refractory or toxic end-of-life products. These urban activities account largely for the biodiversity reduction and the shortage of agricultural and animal husbandry (AG and AH) resources and products [1–3]. Adopting appropriate methods to ensure the safe production of AG and AH products has become one research hub of academia. It also aims to improve product quality while accelerating the distribution efficiency of AG and AH products [4]. Many defects undermine the traditional AG and AH production parks, such as low efficiency and productivity and below-than-average management level [5]. Additionally, many AG and AH parks adopt decentralized data management, making it hard to ensure product safety and quality. Fortunately, technological updates have provided new solutions for traditional AG and AH parks through smart production. Accordingly, the present work is motivated to propose an AG and AH-oriented intelligent production system using a lightweight deep reinforcement learning (DRL) model based on the concept of circular symbiosis [6]. Smart devices can improve the management efficiency of production parks while reducing costs [7].

Many scholars have researched the deep learning (DL) model and the construction of AG and AH production parks. Kamilaris and Prenafeta-Boldú [8] investigated the DL model in agriculture. They analyzed the current situation, advantages, disadvantages, and potential of advanced agricultural DL in various agricultural problems. Zhang et al. [9] applied DL in agriculture-intensive scenario analysis, providing important reference value for developing agriculture. Wang et al. [10] reviewed the application of DL in agricultural hyperspectral image analysis. They summarized maturity, component prediction, different classification topics, and plant disease detection. The results showed that compared with traditional machine learning (ML), DL technology improved the performance of hyperspectral image analysis. According to the above literature, the DL model has important application value in crop classification, detection, counting, and yield estimation in AG and AH.

According to the concept of circular symbiosis, the present work uses a lightweight DL model to study the landscaping and optimization of AG and AH production parks. The motivation and expected value of the research are to use DRL for landscaping AG and AH production parks. The main contribution and innovation are to build an ecological AG and AH production park under the concept of circular symbiosis. By constructing a circular symbiotic industrial chain, combined with a decision tree algorithm and ensemble learning criteria, the optimal design of the AG and AH production park is studied. A simulation experiment verifies the crop yield performance of the park. The present work is organized as follows. In Section 1, the circular symbiosis and AG and AH landscaping background is introduced. In Section 2, the research focuses on designing AG and AH parks and the lightweight DRL model. Then, following a literature review, the DRL model is deeply explored. In Section 3, the circular symbiosis industry chain is constructed and combined with the ensemble learning criteria and the landscaping and optimization scheme of the AG and AH production park. Further, Section 4 comparatively examines the experimental and control groups' results. Finally, Section 5 draws empirical conclusions. The research has practical reference value for the digital and intelligent development of AG and AH production parks.

## 2. Recent Related Work

### 2.1. Circular Symbiosis Concept and Design of Agricultural and Animal Husbandry (AG and AH) Production Park

Many scholars have researched the concept of circular symbiosis. For instance, Shi and Li [11] studied an industrial ecosystem's sustainable resource utilization strategy based on the symbiosis philosophy. Firstly, they clarified the life cycle (LC) system and symbiosis mode of resource flow. As a result, a symbiosis-based resource flow LC management framework was proposed for the industrial ecosystem. The results showed that the proposed comprehensive evaluation method played an essential part in reducing the environmental impact and promoting sustainable utilization. Wang et al. [12] investigated the dimorphism and circular symbiosis of lichen fungi and corporobacterium multifidus. The findings suggested that the growth regulation of pseudohypha caused by dominant activity mutation might destroy the symbiotic relationship between photo organisms and fungi. Grygorenko et al. [13] dived into the symbiotic relationship between drug discovery and organic chemistry. They evaluated the symbiotic relationship between organic chemistry and drug discovery. The results implied that organic chemistry was the basis of existing and upcoming drugs. The relationship between drug discovery and organic chemistry also affected organic synthesis. Sharma and Sharma [14] analyzed the aboveground and underground circular energy conversion systems. Based on the current ecological evidence, they conducted a future comparative study on sustainable agriculture and the environment. Consequently, the potential operation mechanism was revealed. Wanke et al. [15] inquired about the factors of polysaccharides affecting park microbial interaction. They detected microorganisms by sensing the molecular characteristics of the whole microbial category. The research had practical reference value for supplementing and improving the concept of circular symbiosis.

Numerous research also considers the AG and AH production park landscaping. Shi et al. [16] constructed the heterogeneous AG and AH ecotone in the upper reaches of the Yellow River. They determined the ecological source area and resistance surface using morphological spatial pattern analysis and structural equation model (SEM). Meanwhile, Graphab was used to construct the ecological network. The numerical results implied that grazing and agricultural activities were the main reasons underlying the landscape complexity and heterogeneity. The study also revealed the interaction between geographical conditions and landscape heterogeneity. Liu [17] landscaped and researched agricultural theme parks based on ecological-friendly, sustainable development, and agricultural economy philosophy. The research analyzed the background requirements and public needs of agricultural theme park landscaping. Consequently, the proposed landscaping method had a particular effect. It provided a reference value for the subsequent agricultural theme park landscaping. Lavorel et al. [18] studied the multi-functional landscaping template. They systematically analyzed the impact of space fragmentation on landscape versatility and its interaction with land-use intensity. The outcome was that extensive grassland and space supported multiple ecosystem services in the heterogeneous landscape with medium land-use intensity. Increasing land-use intensity would reduce the versatility of the ecosystem while reducing ecosystem services.

### 2.2. Recent Related Work on Lightweight Deep Reinforcement Learning (DRL) Model

In terms of the literature on the lightweight DRL model, many scholars have utilized such technologies as unmanned aerial vehicle (UAV) and mobile edge computing (MEC). To name a few, Lu et al. [19] explored the MEC technology based on DRL to optimize the lightweight task unloading strategy. Specifically, they studied the unloading problem in multiple service nodes and the multi-dependences of mobile tasks in a large-scale heterogeneous MEC environment. The research was based on the improved DRL and candidate networks on long short-term memory (LSTM). The simulation results proved that the proposed improved deep reinforcement Q-learning (IDRQN) algorithm outmatched other algorithms in terms of energy consumption and average execution time. Chen et al. [20] summarized the existing network security based on DL technology. They classified the DL model-based network security applications. The results found that the DL-based correct design method was critical to protecting the smart city network. Lv et al. [21] designed and studied the new system in the smart city vertical market from an economic perspective. The experimental outcomes proved the security performance of the constructed system in six different channels. Further, simulation analysis presented that the constructed system had good stability. From the vertical market perspective, self-operated retailers had more advantages [21]. Thus, the findings can provide an experimental reference for constructing a smart city and developing economy. Li et al. [22] researched medical image fusion methods by DL technology. The research emphasized the information clarity of multi-modal images. Then, the experiment realized the multi-modal medical image fusion based on supervised DL. The proposed method achieved the most advanced performance in visual quality and quantitative evaluation indicators.

Additionally, the research on the DRL model and adaptive DRL (ADRL) model is also the focus of scholars. Huang et al. [23] studied the loop filter based on ADRL and proposed a loop filter construction method for general video coding. Then, they used the latest progress of DRL to select the appropriate network. Extensive experimental results showed that the proposed method could achieve an average efficiency of 2.58% and 2.51% under low latency and random-access configuration, respectively. Meanwhile, the proposed method outperformed other DL methods under low computational complexity. Lu et al. [24] investigated the online tracker selection based on DRL. Through the real-time segmented target tracking strategy in the traffic scene, they studied how to capture and track the environment's dynamics effectively. Also, they regarded the tracker selection problem as a partially observable Markov decision-making process. The results discovered that the proposed tracker had superior performance and achieved an excellent balance between accuracy and efficiency than other state-of-the-art methods.

To sum up, scholars' research on lightweight DRL models has involved many fields: energy management, intelligent monitoring, and Internet of Things (IoT) security intrusion detection. Accordingly, an efficient two-tier optimizer can simultaneously select the input features, training examples, and the number of hidden neurons to implement the DRL model on IoT devices such that the lightweight deep autoencoder model is updated. Then, the optimized DRL model is implemented using the highly accurate K-nearest neighbors (KNN) classifier and the complex autoencoder model. As a result, the model accuracy and efficiency can be both improved. However, the research on the DRL model for landscaping the AG and AH production park is relatively scarce. Therefore, using the concept of circular symbiosis to guide the implementation of the lightweight DRL model and design and improve the production plants can further improve the output efficiency of AG and AH products.

## 3. Landscaping Method of AG and AH Production Park Based on Lightweight Deep Reinforcement Learning (DRL) Model

### 3.1. Construction of Circular Symbiosis Industry Chain

**Figure 1:**
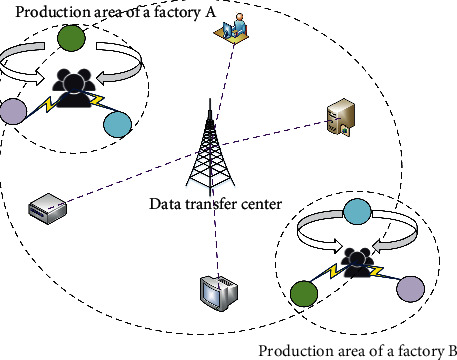
Circular symbiosis system in ecological production park.

With the rapid socioeconomic development, the AG and AH industry has to upgrade towards a higher industrial level based on the regional economy. Meanwhile, it has to improve its resource utilization, reduce energy consumption and environmental pollution, and coordinate regional economy and environmental protection [25]. In constructing the circular symbiotic chain of the ecological industry, the main steps are as follows. Firstly, the related concepts are reviewed on ecological agriculture, agricultural industry, biological symbiosis, and food chain network. Then, it realizes the regional joint landscaping based on environmental protection, ecological diversity, functional positioning, vertical extension, and horizontal coupling of core industries. Finally, through the waste recycling of workshops in the production park, a circular symbiotic industrial system with unified social and ecological benefits is realized. In the whole process, the industrial data are collected from two ecological parks, A and B, and sent to the user for viewing. The circular symbiosis system of the production park is shown in Figure 1.

### 3.2. Decision Tree (DT) Algorithm and Ensemble Learning Criterion

**Figure 2:**
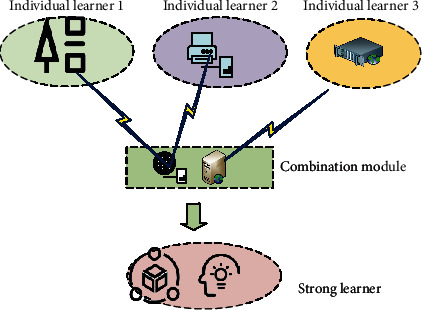
Ensemble learning optimization model based on parallel evolution method.

The DT algorithm uses a series of constraint criteria in the DRL model to classify and judge the data. Then, it generates understandable rules based on inductive criteria to classify and sort data by approximating the discrete function. DT algorithm construction process is generally divided into three steps. First, split the internal nodes by selecting the most critical features from the training data features. Second, the DT is generated through the training sample set. Third, through continuous data division, select training samples closer to the correct classification data set and constantly prune and improve the performance of the DT. Additionally, learning tasks can be generated through individual learners by constructing and assembling multiple learners. The same kind of neural networks can be assembled and optimized through homogeneous integration. Finally, resource allocation and model supervision can be effectively solved through the organic combination of learners. Different individual learners enter the system combination module through a wireless connection and integrate and optimize the overall learning ability. The ensemble learning optimization model based on the parallel evolution method is given in Figure 2.

### 3.3. Landscaping and Optimizing Agricultural and Animal Husbandry (AG and AH) Production Park

**Figure 3:**
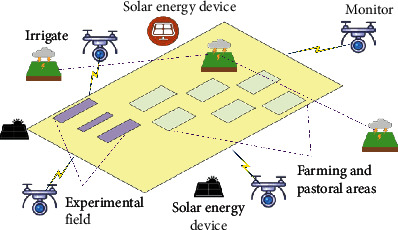
Structure of AG and AH production park.

AG and AH production park is a necessary environment for the growth and maturity of products in the ecological experimental park. The AG and AH production park can intuitively reflect the overall industrial level based on the lightweight DRL model. In particular, landscape elements based on crop planting and ecological landscape concepts, such as permeable pavement and green planting, can further highlight ecological protection and production coordination. For example, permeable pavement can help rainwater penetrate the ground and soil, reduce surface runoff, and improve irrigation. On the other hand, green planting can create a rich ecological landscape, beautify the environment, generate oxygen, and purify the suspended particles. In addition, the development and utilization of solar energy can provide sufficient light, shelter, and pavement. Solar energy can also be stored during the day and then slowly released at night to illuminate crops. This ensures the temperature and light conditions for crop growth in the park. The establishment of the AG and AH production park is sketched in Figure 3.

### 3.4. Experimental Verification

**Figure 4:**
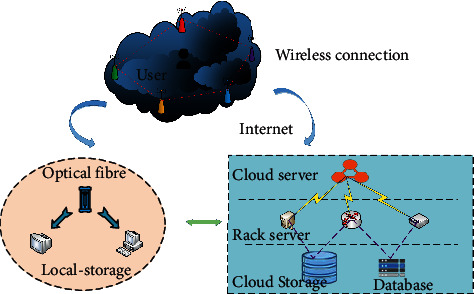
Experimental design and verification environment.

The experimental site is based on the crop irrigation and production park with flat terrain of 12 square meters. Before planting experimental crops, 80 cm wide plastic cloth is used to isolate the experimental area from the rest of the AG and AH production park. Small intelligent sensors are placed in densely crop populated areas to collect real-time meteorological data. It is planned to collect the water content data at the soil depth of 40 cm, and the average error tolerance is within 0.05%. Additionally, after the landscaping and optimization based on DRL, the performance of crop water storage, yield, survival rate, and other parameters have changed significantly. Further, to compare the experimental performance parameters of different crops before and after model optimization, the crop data are extracted from the AG and AH production park and experimental field. Then, the experimental parameters before and after model optimization are compared from crop water storage, yield, and survival rate. Six crops (rice, wheat, corn, potato, tomato, and cucumber) are selected for the experiment, combined with the soil geological conditions of the experimental area and the production park. The data collected from the production park refer to the experimental group, and the crop data extracted from the experimental field refer to the control group. Two groups of data of the experimental and control groups are set for experimental verification. The system data acquisition module is used to collect data, including air pressure, light, and PM2 5, soil temperature and humidity, and air temperature and humidity data. The wireless monitoring network adopts STC12C5A60S2 single-chip microcomputer as the core, working under 5.5 V and 0–35 MHz. 1,280-byte random access memory (RAM) is integrated into the sensor chip, and the detection error rate is 3%. The experimental environment is designed based on Internet, local storage, and cloud storage. Users can easily receive data from the cloud server and rack server port through the wireless network connection. The experimental design and verification environment are illustrated in Figure 4.

## 4. Results and Discussion

### 4.1. Comparison of Crop Water Storage between Experimental Group and Control Group

**Figure 5:**
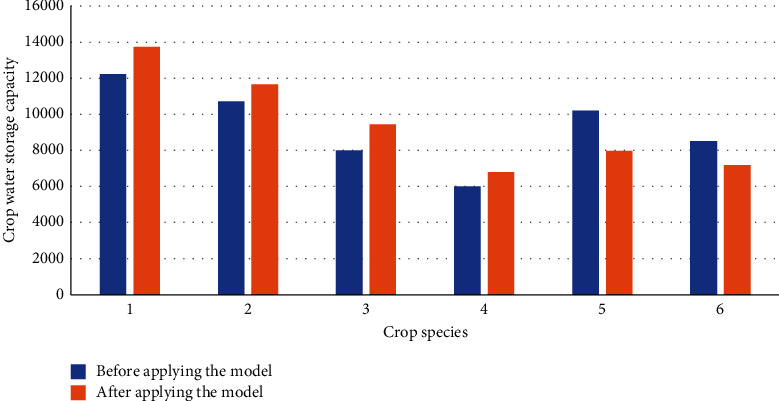
Water storage of different crop species in AG and AH production park before and after model optimization (1, rice; 2, wheat; 3, corn; 4, potato; 5, tomato; 6, cucumber).

**Figure 6:**
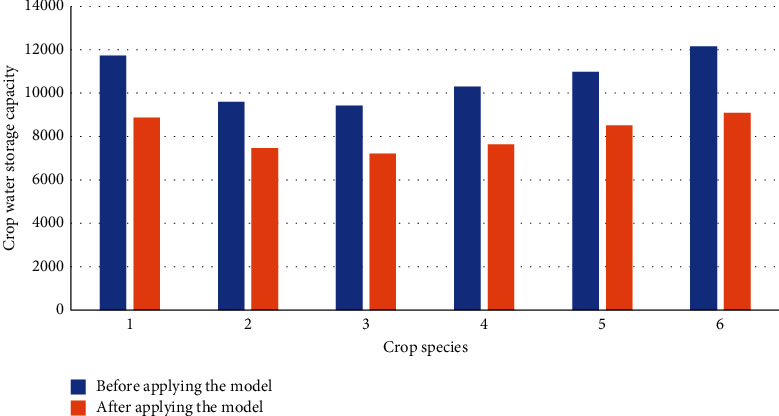
Water storage of different crop species in the experimental field before and after model optimization (1, rice; 2, wheat; 3, corn; 4, potato; 5, tomato; 6, cucumber).

This section compares the performance of the AG and AH production park before and after model optimization based on crop water storage. Thus, the crop data are extracted from the production park and the experimental field. Then, they are divided into the experimental group (crop data of the AG and AH production park) and the control group (experimental field data). Precisely, the control group measures data of the same crop in the experimental field. Figures 5 and 6 give the data statistics.

From Figure 5, the landscaping and optimization of the AG and AH production park using the DRL model have improved the crop water storage of rice, wheat, corn, and other crops to a certain extent. Among them, the water storage of rice and corn is significantly improved, so these two crops are suitable for planting in the optimized production park. By comparison, the water storage of tomato and cucumber has shrunk after optimization, so these two crops are more suitable for centralized planting using the traditional planting method. The optimized production park will weaken the water storage of the tomato and cucumber.

From Figure 6, compared with the AG and AH production park, the water storage of several crops in the experimental field diminishes to a certain extent after model optimization. Noticeably, the water storage reduction of cucumber is the most obvious. The water storage of cucumber can reach 12,000 before the model optimization and drops to about 9,000 after optimization, with a significant reduction. Additionally, the water storage of the other crops also declines to different degrees. Hence, the improved crop planting method is unsuitable for these crops in the experimental field. Here, the traditional planting method might achieve better results.

### 4.2. Comparison of Crop Yield between Experimental Group and Control Group

**Figure 7:**
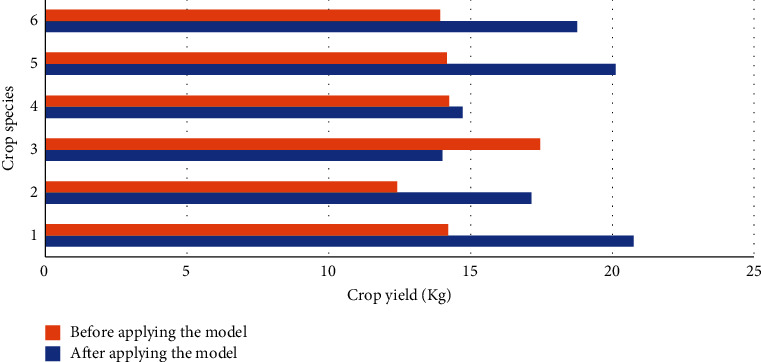
Crop yield of different crop species before and after model optimization in the AG and AH production park (1, rice; 2, wheat; 3, corn; 4, potato; 5, tomato; 6, cucumber).

**Figure 8:**
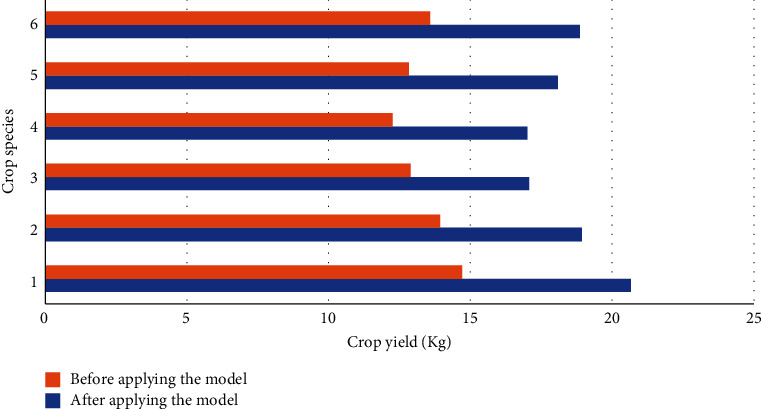
Crop yield of different crop species in the experimental field before and after model optimization (1, rice; 2, wheat; 3, corn; 4, potato; 5, tomato; 6, cucumber).

This section examines the landscaping performance of the lightweight DRL model in optimizing the AG and AH production park based on crop yield. Then, the experimental and control groups collect the crop yield data of different crops in the AG and AH park and the experimental field before and after the model optimization. The data are detailed in Figures 7 and 8.

From Figure 7, the yield of several crops has significantly improved after model optimization. More precisely, the yield improvement of rice is the most prominent. For example, the rice yield in the AG and AH production park is less than 15 kg before model optimization and over 20 kg after optimization. Besides, wheat, potato, tomato, and cucumber yield has increased to varying degrees. In contrast, the corn yield has decreased after the model optimization, so the optimized production park environment is not suitable for planting this variety of corn. It is suggested to reduce the planting area of this variety of corn in the optimized production park.

From Figure 8, the yield of various crops has effectively improved in the experimental field. For example, yield improvement of cucumber, wheat, and rice is the best, only about 12 kg before optimization and 18 kg after DRL-optimized production park landscaping. Thus, the proposed DRL-based production park landscaping is proven to be reliable and effective.

### 4.3. Comparison of Crop Survival Rate between Experimental Group and Control Group

**Figure 9:**
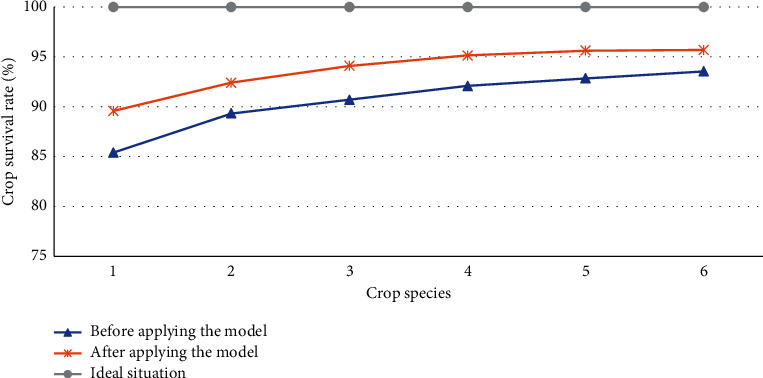
Crop survival rate of different crop species before and after optimization in the AG and AH park (1, rice; 2, wheat; 3, corn; 4, potato; 5, tomato; 6, cucumber).

**Figure 10:**
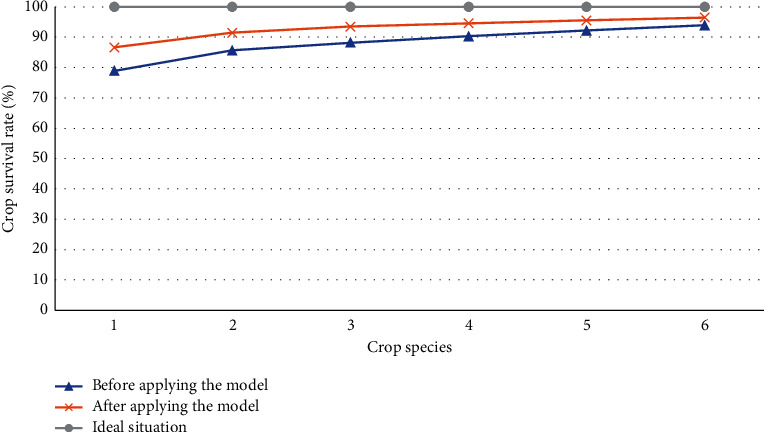
Crop survival rate of different crop species in the experimental field before and after optimization (1, rice; 2, wheat; 3, corn; 4, potato; 5, tomato; 6, cucumber).

Among the many measurement data in the AG and AH production park, the crop survival rate can reasonably evaluate the performance of the DRL model optimization. Therefore, Figures 9 and 10 compare the crop survival rate before and after the production park optimization based on the DRL model.

From Figure 9, the survival rate of all crops after DRL model optimization is closer to the ideal situation rate (100%). Noticeably, the survival rate of rice is improved the most, about 85% before optimization and up to 90% after. Moreover, the survival rate of cucumber is the best among several crops, 94% before optimization and 95.4% after, improved by 1.4. Therefore, the lightweight DRL model can effectively optimize the crop planting conditions in the overall AG and AH production park and improve their survival rate.

From Figure 10, the survival rate of several crops has declined somehow in the experimental field compared to that within the overall AG and AH production park. For example, in the experimental field, the initial survival rate of the crop is only 80% and nearly 90% after optimization. Furthermore, the survival rate of wheat, corn, potato, and tomato in the experimental field was not very good initially. Nevertheless, the DLR model can increase their survival rate by about 3.5%. At the same time, the DLR model has not significantly improved the survival rate of cucumber, only from 94.7% to 95.3%. To sum up, in the lightweight DRL-optimized AG and AH production park, the survival rate has risen, much closer to the ideal one.

## 5. Conclusion

Under the concept of circular symbiosis, to improve the output efficiency of AG and AH products, it is necessary to optimize AG and AH production park landscaping. Starting from reality, based on the construction criteria of circular symbiotic industrial chain, DT algorithm, and DRL, the present work studies the landscaping and optimization scheme of AG and AH products. At the same time, it compares the experimental and control groups' numerical values. The experimental results suggest that after the DRL-optimized AG and AH production park landscaping, the water storage of rice, wheat, corn, and potato has been improved to a certain extent. Remarkably, the water storage of rice and corn is significantly improved. On the other hand, rice yield improvement is the most obvious, with less than 15 kg in the AG and AH production park before optimization and over 20 kg after. Finally, there are still some deficiencies, mainly because few crop types are selected in the experimental field, which is insufficient to generalize the research results. Future research expects to supplement and enrich the crop types, optimize the model functions, and expand the application prospect of the lightweight DRL model. The result has practical application value for improving crop yield in agricultural production parks.

## Data Availability

The data used to support the findings of this study are included within the article.
